# Flavouring Tunisian Extra Virgin Olive Oil (EVOO) with Cloves: Quality Indices, Stability, and Consumers’ Purchase Survey

**DOI:** 10.3390/foods14122114

**Published:** 2025-06-16

**Authors:** Monia Ennouri, Slim Smaoui, Theodoros Varzakas

**Affiliations:** 1 Olive Tree Institute, University of Sfax, Road of Airport Km 1.5, P.O. Box 1087, Sfax 3000, Tunisia; monia.ennouri@enis.rnu.tn; 2 Higher Institute of Applied Science and Technology of Mahdia, University of Monastir, Road Rejiche, Mahdia 5121, Tunisia; 3 Laboratory of Microbial Biotechnology and Engineering Enzymes (LMBEE), Center of Biotechnology of Sfax (CBS), University of Sfax, Road of Sidi Mansour Km 6, P.O. Box 1177, Sfax 3018, Tunisia; slim.smaoui@cbs.rnrt.tn; 4 Department of Food Science and Technology, University of the Peloponnese, Antikalamos, 24100 Kalamata, Greece

**Keywords:** Tunisian olive oil, cloves, quality, stability, consumer survey

## Abstract

The objective of our study is to monitor the stability of Extra Virgin Olive Oil (EVOO) flavoured with cloves. Two flavouring processes were tested, namely the maceration of cloves in olive oil and the grinding of cloves with olives. The analysis of the obtained oils showed that the process of the simultaneous grinding of the cloves with the olives produced a better oil quality than the maceration process in terms of richness in total phenols. The co-crushing method increased the total phenols in the olive oil by 34.24% and 73.37%, compared to the maceration method with an increase of only 17.1% and 52.35%, respectively, for the 2 and 4% of cloves addition. Fluorescence spectroscopy analysis of the oils supplied useful and complementary results. The aromatized olive oil developed by simultaneous grinding was subjected to ageing acceleration at 60 °C in the dark for 165 days. Results indicated that the acidity and the value of the specific extinction coefficient K_232_ of the control EVOO followed the standards of the International Olive Oil Council. During accelerated storage, the degradation of total phenols was marked as less for the flavoured EVOOs than for the control samples. After 165 days of storage, the colour of all olive oil samples was modified, with this change being the most apparent for unflavoured oil with a 45.6% and 46.4% decrease in L and b* vs. 38.8% and 22.4% for C1, and 45.5% and 37.2% for C2 respectively. After 165 days of storage, all the oil samples were darker and red. Flavouring EVOO with cloves offered a better stability to the oil. A consumer survey involving 224 participants revealed that despite the fact that only 30% were familiar with flavoured oils, 83.9% expressed a willingness to purchase clove-flavoured olive oil if it became available on the market. Flavoured oils offer a good alternative to multiply olive oil-based products and thus offer additional opportunities for the marketing of olive oils.

## 1. Introduction

Olive oil is certainly one of the pillars of the Mediterranean diet combining consumer satisfaction and benefits for human health [1]. The richness of olive oil in polyunsaturated fatty acids, polyphenols and antioxidants makes it an ally preventing several diseases. The effects of olive oil on cardiovascular disease, prevention of cancers and many other diseases have been well studied and validated due to the existence of many beneficial elements in oil (hydrotyrosol, tyrosol and oleuropein, etc.) [2,3]. Recent studies reported that the consumption of olive oil by the elderly has benefits on cognitive performance, promoting the increase in cognitive functions and the reduction in their decline [4,5,6]. Olive oils differ in their composition, their taste and the richness of their aromas according to the ripening, the variety, the environmental conditions, the soil compositions and oil processing extraction techniques [7,8,9]. According to the International Olive Council, three classes of olive oil, fit for consumption, exist: ordinary olive oil, virgin olive oil and extra virgin olive oil [10]. All oil categories can be flavoured depending on their purpose. Flavoured olive oils, called also “gourmet oils”, can increase demand for olive oil from consumers who are looking for new tastes and aromas while enjoying the advantages of olive oil, to which the properties of flavouring agents are added.

The flavouring of olive oil can have different goals. In fact, a flavouring agent can mask a defect (undesirable odour or taste), reduce the intensity of an attribute naturally present in the oil (bitterness, astringency, spiciness or other), enhance the flavour of a flat oil lacking fruitiness, or add a new aromatic bouquet to an extra virgin olive oil that is free of defects. For aromatization process, medicinal and aromatic plants as well as fruits and vegetables are used in fresh or dried form or in extracts like essential oils [11,12]. Different procedures of incorporation of aromatic agents exist, like maceration, incorporation of essential oils and co-extraction [13]. Accordingly, the choice of a suitable technique is critical, given that the aromatization process may impact the sensory acceptance and oxidative stability of the oil [14,15].

Maceration or infusion is a traditional method that involves bringing finely ground-flavouring materials into contact with extracted olive oil. The mixture is left at room temperature, and regularly stirred to enable the diffusion of the flavouring compounds [14]. The combined mixing of olive paste with aromatic plants involves the direct addition of crushed and/or whole plant materials to the olives or olive purée during the crushing and malaxation stages [11,16], hence allowing the better migration of aromas and bioactive antioxidant compounds that are generally hydrophobic from the flavouring agent to olive oil [17]. For instance, Habibi et al. [18] have reported the improvement of the quality of olive oil obtained from fallen and ripe olives by incorporating rosemary during the olive crushing process. In comparison to virgin olive oil, the flavoured oil exhibited an improved sensory profile, along with higher polyphenol content and enhanced antioxidant activity. Aromatization improves the stability of oil toward oxidation, which can be assessed through quality standards like peroxide value, free fatty acids and specific extinction values.

Clove (*Syzygium aromaticum*), a plant belonging to the Myrtaceae family, is used in pharmaceutical applications, cosmetics, culinary preparations, and active packaging due to its antibacterial, antiseptic, anticarcinogenic, and antioxidant properties [19,20]. The clove is used as a spice in traditional dishes for aromatization, as a preservative against foodborne pathogens and as a natural colourant in food preparations [21,22]. Recently, clove extracts were successfully used added to a chocolate beverage [23], in meat preservation [24,25], in wheat to control wheat common bunt, in baked food for preservation [26], in dairy products [27] and many others. According to the World Health Organization (WHO), the permitted daily amount of clove for human consumption is 2.5 mg/kg body weight [28].

To the best of our knowledge, the only study where flavoured olive oil with cloves was used was carried out by Trabelsi et al. [29]. They have macerated the cloves in olive oil at a concentration of 50 g/kg, at ambient temperature, for 30 min. The aim of the study was to compare different flavoured oils for their potential as anisakicidal agents in the industrial process of marinating anchovies.

The purchasing and consumption habits of Tunisians for olive oil and aromatic oils are not yet documented; in fact, no studies dealing with this subject were found. In their review of olive oil preferences and consumer acceptance, Latino et al. [30] noted the lack of studies on Tunisian consumers. This highlights the importance of understanding and exploring their purchasing habits, preferences for olive oil, and perceptions of oils displayed in supermarkets.

The objectives of this study were to (1) compare the aromatization techniques, maceration vs. co-crushing at two concentrations of cloves in EVOO, (2) study the ageing of the flavoured EVOO with cloves during 165 days at 60 °C, and (3) evaluate the knowledge of consumers concerning the flavoured olive oil with cloves and their intention of purchase.

## 2. Materials and Methods

### 2.1. Oil Flavouring

For the present study the variety of olive tree “Chemlali Sfax” was used (from the region of Sfax). Olives were harvested in November 2023. The olives were harvested at a maturity index of 4, fruit maturity was assessed based on the skin and pulp colour of 100 olives, using a scale from 0 to 7, where 0 indicated completely dark green skin and 7 indicated fully black skin with purple flesh. The extraction of the olive oil was performed in several stages. First, the olives were sorted to remove leaves, twigs, small stones and soil. Then they were immediately transported to the laboratory for extraction on a lab-pilot oil extractor. The sorted olives were washed under running water and then crushed. This step aims to break down the olive cells and release the oil droplets stored in the vacuole. During the mixing stage (at ambient temperature during 40 min), an important operation to increase extraction yield, the aggregation of oil droplets are promoted in order to form larger ones. After a centrifugation step, the obtained olive oil underwent decanting for 24 h in the dark. Finally, the oil was stored in opaque bottles, at a controlled temperature, away from air and light to preserve its freshness, taste and fruity scent, and to avoid oxidation. The cloves were purchased from a spice store and were crushed with a domestic crusher (Moulinex, France).

The impact of adding cloves on olive oil stability was evaluated. Two clove concentrations were tested: C1, which corresponds to 2% (*w*/*w* cloves/oil), and C2, which corresponds to 4% (*w*/*w* cloves/oil). These were compared to C0, the control oil (unflavoured EVOO). The way of flavouring the oil was one of the main concerns of this study. Two different flavouring processes were tested. For the maceration method, clove powder was added to EVOO at two concentrations, 2% (AOMC1) and 4% (AOMC2), and the mixture was agitated for 2 h at ambient temperature. Then the mixture was kept in opaque glass bottles for 20 days and after that filtered to remove plant material. For the co-crushing method, the clove powder was added directly in the crusher (at two concentrations, C1 and C2). For each oil type, three independent samples were analysed.

### 2.2. Oil Ageing Test

Seventy grams of each oil sample were placed in 30 mL capacity flasks and stored in an oven set at 60 °C (Binder, Model No: 970465, Tuttlingen, Germany). These elevated temperature conditions were used to accelerate the ageing process, simulating long-term storage at room temperature. At predetermined time intervals over a total period of 165 days, individual samples were removed from the oven for analysis. The primary aim of this thermal treatment was to evaluate the oxidative stability of the oils under accelerated conditions. To monitor the progression of oxidation and quality deterioration, several parameters were measured: acidity (as an indicator of hydrolytic degradation), specific extinction coefficients at 232 nm and 270 nm (K_232_ and K_270_, markers of primary and secondary oxidation products), as well as the concentrations of carotenoids, chlorophylls, and total phenolic compounds, which are important natural antioxidants in oils.

### 2.3. Determination of Oil Parameters

#### 2.3.1. Density

The density of the oil, which is an indicator of its purity, was measured according to Wolf procedure [31].

#### 2.3.2. Quality Indices

Free fatty acids (FFAs), peroxide value (meq O_2_/kg oil) and spectroscopic indexes (K_232_, K_270_) were established based on the official methods outlined by the International Olive Council [32,33,34].

#### 2.3.3. Pigments Quantification

Carotenoids and chlorophylls contents (mg/kg oil) were measured spectrophotometrically in cyclohexane at 470 nm and 670 nm, respectively [35].

#### 2.3.4. Colour Determination

Olive oil colour was assessed with a spectrophoto–colourimeter (Trintometre, Lovibond PFX 195 V 3.2, Amesbury, UK) and represented by chromatic ordinates L, a* and b*, respectively, for lightness, redness, and yellowness [36].

#### 2.3.5. Total Phenols Determination

Total phenolic contents were determined according to the method described by Ammar et al. [36] and expressed as milligrams of gallic acid equivalent (GAE) per kg of oil.

#### 2.3.6. Fatty Acids Determination

The fatty acid composition of oils was analysed via gas chromatography (GC) (Shimadzu 17A gas chromatograph (Kyoto, Japan) equipped with a flame ionisation detector (FID) and a capillary column) as fatty acid methyl esters [37].

#### 2.3.7. Fluorescence Spectroscopy

Fluorescence spectra were studied using a Fluoromax-4 spectrofluorimeter (Jobin Yvon, Horiba, NJ, USA) at 20 °C. The excitation radiation incidence angle was set to 60° to minimise reflected light, scattered radiation, and depolarization effects. For each oil sample, 3 mL were placed in a quartz cuvette, and fluorescence spectra were recorded. The emission spectra for polyphenols (290–450 nm) and chlorophylls (450–800 nm) were obtained with excitation wavelengths set at 270 nm and 430 nm, respectively. Three spectra were recorded for each sample.

### 2.4. Consumer Survey

In order to study the interest of consumers for flavoured olive oil, a consumer survey was conducted on a sample of 224 Tunisian consumers of different categories and ages. It was divided into four themes: social situation (age, gender, educational level), the mode of consumption of olive oil (how they buy their olive oil, how often they use olive oil, what is their monthly consumption of olive oil, the manner in which they use olive oil), their interest in cloves and flavoured olive oil, as well as their intention to purchase clove-flavoured olive oil if it becomes available on the market. The survey was planned using Google forms and shared through social networks.

### 2.5. Statistical Analysis

The physicochemical analyses were conducted in triplicate. The results were presented as mean value ± standard deviation (SD). SPSS statistical software (Chicago, IL, USA) version 16.0 was employed to analyse the data. Duncan’s multiple range post hoc test was applied in a two-way analysis of variance (ANOVA) with SPSS at a 95% confidence level (*p* < 0.05) to find variations among samples. PCA were performed using XLSTAT 2016 (Addinsoft SARL USA, New York, NY, USA).

## 3. Results and Discussion

### 3.1. Effect of Aromatization on the Physicochemical Characterisation of Flavoured EVOOs

The two extraction methods, namely co-extraction and maceration, were performed with two concentrations of cloves, at 2% and 4%. The physicochemical parameters of the oils are shown in Table 1. The density of the oil was affected by aromatization since density increased with aromatization. The colour attributes L (light–dark), b* (yellow–blue) and a* (red–green) were determined (Table 1). The incorporation of cloves in olive oil significantly decreased the luminance (L) at (*p* < 0.05), ranging from 21.81 for control to 18.37 for C1. The decrease in luminance was more evident with maceration (AOMC1 and AOMC2) compared to co-crushing (C1 and C2). Flavouring also significantly decreased b* (*p* < 0.05), specifically with maceration (2.483 for AOMC1 versus 11.39 for C1), while a* increased significantly when the concentration of cloves in the olive oil increased. The same observation was reported by Aljobair [38] when adding clove powder (at 2%) into the formulation of cookies—they reported a decrease in L and a* in the supplemented cookies compared to control. Flavouring significantly altered the colour of the EVOO, with the variation being more pronounced when the flavouring process was conducted via maceration compared to co-crushing. This is likely due to the longer diffusion period of colour pigments in maceration (20 days), whereas co-crushing occurred over just a few hours. Flavouring with cloves notably enriched the EVOO with pigments, resulting in a darker, less green, and more reddish hue. Cloves contain dark-coloured pigments like tannins and other polyphenols (e.g., eugenol), which can leach into the oil during maceration, altering the colour. The longer extraction time during maceration allowed for more of these pigments to dissolve and affect the final oil colour.

The free fatty acid contents and peroxide value increased significantly when the EVOO was flavoured by maceration, leading to a less stable oil. The control EVOO presented specific extinction coefficients (K_232_ and K_270_) in agreement with the IOC standard for extra virgin olive oil (K_232_ ≤ 2.50 and K_270_ ≤ 0.22), whereas in flavoured EVOO only AOMC2 showed an increase in K232. For K270, AOMC1 and AOMC2 showed an increase and exceeded the limit. Despite having no standards for flavoured EVOOs, different studies determine them similarly (e.g., Diaz-Montana et al. [39] have used these methods for olive oil flavoured with rosemary and basil herbs and Revelou et al. [40] have used the same methods for *Origanum majorana* L.-aromatised EVOO).

In fact, flavoured olive oils can derive from one of the three categories of olive oil, but the aromatization process makes them different. K_232_ and K_270_ are associated, respectively, to the presence of primary and secondary oxidative products. A variation in these parameters with flavouring was shown. The increase in K_270_ in flavoured EVOO samples is not in accordance with the effect expected by the addition of the flavouring agent, which is to enhance the oxidative stability of the oil, and so it is assumed that we should observe a significant decrease in K_270_. Gambacorta et al. [41] also reported an increase in K_270_ in olive oil flavoured with rosemary, garlic, pepper, and oregano. Similarly, Sacchi et al. [42] observed the same trend in olive oil flavoured with lemons, attributing the increase in K_270_ to the presence of terpenes in the flavouring agent, which absorb in the 232–270 nm wavelength region. These substances diffuse from the flavouring agent to the oil, interfering with the signal.

Chlorophyll content was significantly more important in co-crushing (4.12 and 4.37 mg/kg, respectively, for C1 and C2) compared to maceration (2.71 and 1.98 mg/kg, respectively, for AOMC1 and AOMC2) (*p* < 0.05). This was probably due to the degradation of chlorophylls during maceration. Carotenoids changed during aromatization—increased with co-crushing and decreased with maceration.

The increase in carotenoids with flavouring was in accordance with the variations observed previously in colour attribute a*. The total phenol content of the control sample (CO) was 385.7 mg GAE/kg, whereas it was estimated to be equal to 517.88 and 451.62 mg GAE/kg for the EVOO with 2% cloves (C1), with co-crushing and maceration, respectively. The highest total phenol content was reported in the EVOO with 4% of cloves, flavoured using the co-crushing process, followed by the oil containing the same concentration of cloves, flavoured using the maceration method. The co-crushing method increased the total phenols in the olive oil by 34.24% and 73.37% for 2% and 4% of supplementation, respectively.

### 3.2. Fatty Acid Analysis

The fatty acid analysis of control and flavoured EVOO is shown in Table 2. The principal fatty acids were shown to be oleic acid (60.4%) followed by palmitic acid (18%) and linoleic acid (15.8%). These percentages agree with those cited in the reports for the variety Chemlali Sfax [43]. A slight increase was observed for linoleic acid in flavoured EVOO (15.94%) compared to the control (15.82%). The aromatization of olive oil with cloves had no effect on the fatty acid composition of the oil. Similar results were observed when the oil was aromatized with rosemary [18]. The aromatization with cloves improved the oil with bioactive compounds but not with fatty acids.

### 3.3. Emission Fluorescence Spectra of Polyphenols and Chlorophylls for Aromatized Oils

**Figure 1:**
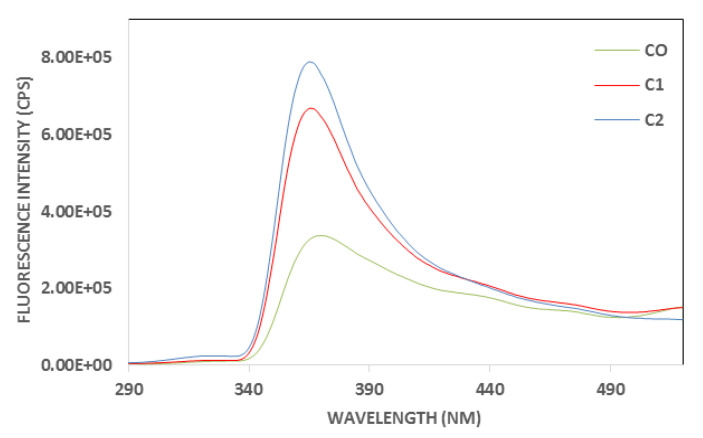
Excitation fluorescence spectra of control (C0) and aromatized oils (C1 and C2) for polyphenols acquired after excitation set at 270 nm.

**Figure 2:**
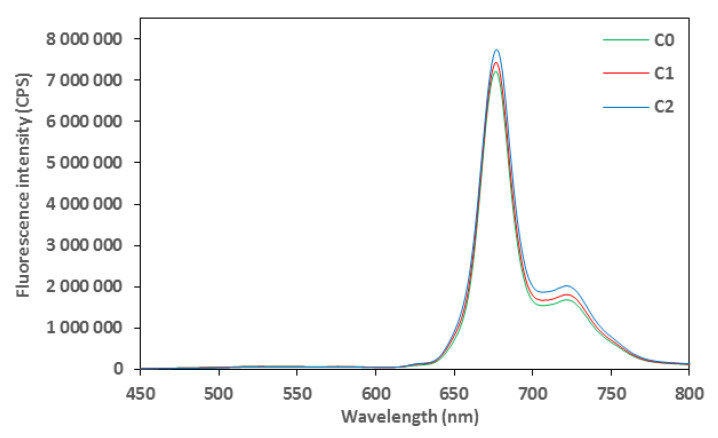
Excitation fluorescence spectra of control (C0) and aromatized oils (C1 and C2) for chlorophylls acquired after excitation set at 430 nm.

Control and aromatized oils were submitted for fluorescence analyses; the results were presented in Figure 1 for polyphenols and Figure 2 for chlorophylls. For the two spectra, the highest fluorescence intensity was noted for C2, indicating a very high antioxidant capacity and chlorophyll content, followed by C1. The control EVOO, C0, showed the lowest fluorescence intensity caused by the lack of supplementation with cloves, which are rich in antioxidant polyphenols and chlorophylls. Indeed, cloves represent one of the major vegetable sources of phenolic compounds such as flavonoids, hidroxibenzoic acids, hidroxicinamic acids and hidroxiphenyl propens. Eugenol is the main bioactive compound of cloves, found in concentrations ranging from 9.38 to 14.65 g per 100 g of fresh plant material [28].

### 3.4. Effect of Ageing on the Quality of Olive Oils

Table S1 shows the evolution of FFAs in flavoured and control EVOOs during storage at 60 °C. The three oils followed the same tendency, so flavouring had no effect on this parameter. The values of FFAs varied from 0.22% at initial time to 3.29% after 165 days of storage.

Concerning the coefficients of extinction for K_270_, flavoured EVOOs had increased values during the first 100 days of storage. Following that, stabilisation of the coefficients was observed at a value of 0.54 (Table S1). For the unflavoured EVOO (control), K_270_ continued to increase until 165 days of storage, at which point it reached 0.702. For K_232_, the values were stable for 130 days of storage; a slight increase was noted for control and flavoured EVOOs with 4% of cloves. The control EVOO, after 165 days of storage at 60 °C, had a value of K_232_ equal to 2.48, which was lower than the limit of the IOC standard for extra virgin olive oil.

Regarding the content of chlorophyll pigments (Table S1), although the control olive oil showed higher contents at the beginning of storage (6.05 mg/kg), these pigments decreased and reached 0.92 mg/kg at 165 days of storage. This decrease could be attributed to the oxidation of the oil induced by storage at 60 °C. For flavoured EVOOs, although the content was lower than that of the control at the beginning of treatment, after 165 days of storage, the content was 1.86 and 1.54 mg/kg, respectively, for C2 and C1. The same tendency was observed for carotenoid content (Table S1), i.e., the values for control oil were lower than those of the flavoured oils at 165 days of storage. It can be concluded that the flavouring of EVOO with cloves aided in the delay of the degradation of chlorophyll and carotenoid pigments.

Flavouring EVOO with cloves significantly increased its total phenolic content (Table S1). Cloves are a rich source of phenolic compounds [44], and while olive oil also contains polyphenols, it does so to a lesser extent. The flavouring of olive oil with clove had an additive effect leading to the enrichment of olive oil with phenolic compounds. Storage led to a decrease in total phenolic in all the oils, the values varied from 668.7 to 382.7 mg/kg for C2, and from 517.8 to 215 mg/kg for C1, and from 385.7 to 215 mg/kg for C0, after 165 days of heat treatment at 60 °C. The EVOO containing the higher concentration of clove (4%) was more stable than the oil containing 2% of clove and control oil. Lazzarini et al. [45] reported the same tendency with co-milling olives with black pepper, orange pomace, and hemp seeds and reported that addition of these matrices increased the amounts of polyphenols and participated in the enhancement of the sensory profile of the oil.

The colour parameters reported in Table 3 indicated that the colour of all the olive oil samples was significantly changed at (*p* < 0.05) and that this change was the most apparent in unflavoured oil, with a 45.6% and 46.4% decrease in L and b* vs. 38.8% 22.4% for C1, respectively. Following 165 days of storage, all the flavoured oil samples were much darker and red, compared to unflavoured oil.

### 3.5. Principal Component Analysis

**Figure 3:**
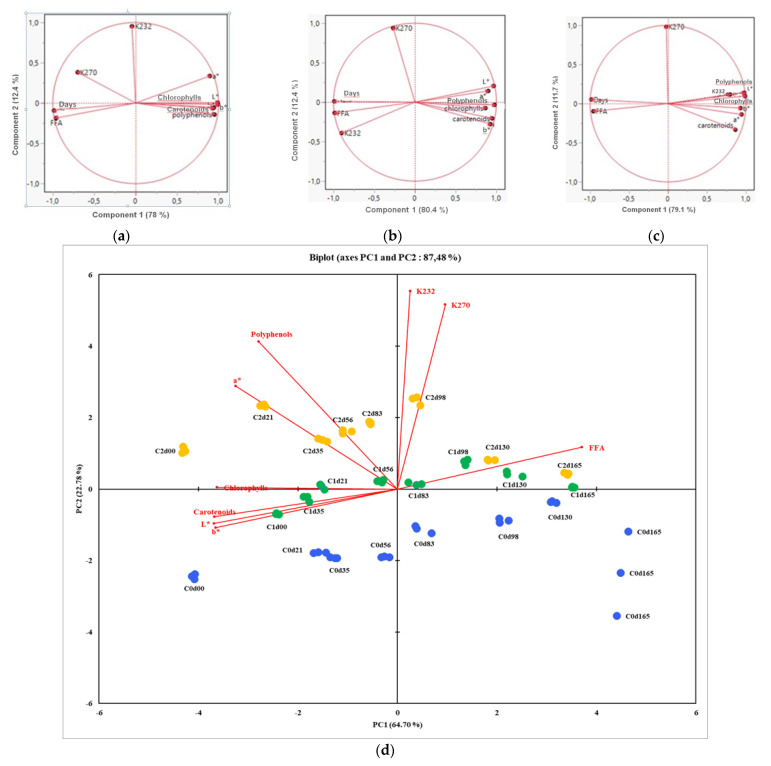
Principal component analysis for (**a**) C0, non-aromatized olive oil, (**b**) C1, aromatized olive oil with 2% cloves, (**c**) C2, aromatized olive oil with 4% cloves, and (**d**), all three samples.

In order to study the correlation between the physicochemical parameters of each oil, a principal component analysis was conducted and is presented in Figure 3.

For the control EVOO non-aromatized with cloves (Figure 3a), the two principal components accounted for 90.4% of the variance, which means that the biplot was a good representation of the data’s structure. Component 1 (78%) is the dominant axis, capturing most of the variation; the variables strongly aligned with this axis contributed most to explaining the differences among samples. Polyphenols, carotenoids, chlorophylls, and colour parameters were positively correlated, whereas FFAs and storage duration were negatively correlated. Polyphenols and chlorophylls were negatively correlated with free fatty acids since they are situated in two opposite directions. Component 1 seems to also represent the oil quality, with the right side of the parameters showing good quality, whereas in the left we found the parameters of deterioration and oxidation of the oil (free fatty acids, K_270_). Component 2 may reflect early oxidation (K_232_) versus later or secondary effects (K_270_).

For aromatized oils (Figure 3b,c) the same trend was observed as for control oil, apart from K_232_ and the K_270_.

Principal Component Analysis (PCA) (Figure 3d) was applied to explore the evolution of olive oil parameters in the three sample groups (C0, C1 and C2) stored over a period of 165 days (Figure 3d). The similarity map of PCA, defined by the first and the second principal components (PC1 and PC2), considering 87.48% of the total variance, showed a clear separation between flavoured and unflavoured olive oil samples based on storage time and associated colorimetric and chemical changes. Indeed, fresh samples (e.g., C0d00, C1d00 and C2d00) are clustered on the left site of the biplot and are closely associated with chlorophylls, carotenoids and colorimetric parameters, reflecting their initial high-quality state. As storage time increased, the olive oil samples shifted progressively towards the right, along PC1, which could reflect an increase in the oxidation markers including FFA, K_232_ and K_270_. This shift could suggest a degradation in olive oil quality during the storage, consistent with the oxidative processes known to occur during storage. Indeed, unflavoured olive oil samples (C0) showed the pronounced changes among other samples, ending near the high oxidative region (e.g., C0d165). Flavoured olive oil C1 follows a similar oxidative path, but at a less intense rate than C0. In contrast, flavoured olive oil samples C2, showed minimal variation across the storage period, indicating better oxidative stability than C0 and C1. Additionally, K_232_, K_270_ and FFA have long arrows pointing to the right which suggests that they are positively correlated with each other and contribute strongly to the increase along PC1. Chlorophylls, carotenoids, colour attributes and polyphenols are oriented to the left, more associated with early storage samples, and are negatively correlated with K_232_, K_270_ and FFA.

Overall, the similarity map of PCA illustrated that storage time alters the chemical and colorimetric profiles of the olive oil samples and highlighted the potential protective effect of flavouring with cloves, especially with C2.

### 3.6. Consumer Survey

The survey was established to study the consumers’ behaviours and their interests in clove-flavoured olive oil. The results showed that most of the participants were under 25 years old and with a high school diploma (51.8%). Little variation was observed in the consumers’ gender level, since the participants included 122 females and 102 males (Table 4). They all signed an ethical consent form before participating in the survey.

The results showed that the majority of consumers buy olive oil directly from the producer (86.5%), and only 4% buy olive oil from a supermarket. It is important to note that Tunisia has 1672 listed olive mills [46]. Olive mills are spread across the region and are easily accessible to buyers. The volumes sold range from ½ litre to several litres, with many consumers preferring to purchase olive oil directly from the mill. When buying from the producer, consumers are given the opportunity to taste different samples and select the oil based on their preferences—whether they favour a mild, sweet flavour or a more intense aroma with a pronounced bitterness.

We found a great lack of awareness of clove-flavoured EVOO, since 70% of participants did not have information about these oils (Table 5). The same trend was observed when the consumers were asked about their doubts in purchasing flavoured olive oil; 22.3% responded that there is a lack of advice in that area, so we can assume that flavoured EVOOs are a niche market. Sixty five percent of participants consumed 2 L or more of olive oil per month, as it is the flagship product of the Mediterranean diet [47]. Concerning the respondents’ hesitancy when buying flavoured oil in the supermarket, 46.6% of consumers had doubts about the quality of the product. Consumers preferred oils that had clear origin information, such as those that are locally sourced. Concerning the purchase intention of flavoured olive oil, 83.9% declared that are ready to buy the clove-flavoured EVOO if they find it on the market, which is very encouraging for the producers of this type of oil.

## 4. Conclusions

Olive oil aromatization is an innovative technique used to create commercial alternatives for olive oil, enhance its quality, and prolong its stability during storage. Cloves have been recognised since the antiquity for their beneficial effects on health and for their antibacterial properties. The aromatization with two concentrations of cloves (2 and 4%) in extra virgin olive oil has been evaluated, along with two methods of aromatization: co-crushing and maceration. According to the results, the best technique for aromatization in terms of polyphenol content increase and quality parameters (PV, FFA, K_232_ and K_270_) was co-crushing. In addition, this technique did not affect the fatty acid composition of the oil. The olive oil aromatized by co-crushing was also studied for its stability against ageing (heating at 60 °C for 165 days). The quality of flavoured and control EVOO was degraded after storage at different degrees, and the more stable oil was that flavoured with 4% of cloves. The nature and concentrations of bioactive substances present in the flavouring agent were crucial for assessing the scope and the rate of the olive oil deterioration after storage at 60 °C. Adding cloves to olive oil enhanced its quality and stability. Moreover, the control olive oil presented specific extinction coefficients (K_232_ and K_270_), in agreement with the IOC standard for extra virgin olive oil (K_232_ ≤ 2.50 and K_270_ ≤ 0.22). For flavoured EVOO, despite it having no official standards, determinations using the IOC standards showed that only AOMC2 showed an increase in K232, whereas for K270, AOMC1 and AOMC2 showed an increase and exceeded the limit.

PCA explained more than 90% of the total variance in the studied oils and revealed a clear dichotomy between freshness-associated parameters (polyphenols, chlorophylls, carotenoids, L *, a*, b*) and degradation-related parameters (free fatty acid). Flavoured olive oil sample C2 showed minimal variation across the storage period, indicating a better oxidative stability than C0 and C1. Hence, it can be concluded that the addition of 4% of cloves the oil showed much better results than 2%. The consumer study revealed that there is a need for more research on the sensory attributes of clove-flavoured olive oil, consumer behaviour and expectations, and the popularisation of flavoured olive oils and their health benefits. Flavoured EVOOs can become even more appealing when marketed as premium products, attracting tourists and becoming central to discovery tours across various Tunisian regions. Each region, distinguished by a unique spice, herb, or fruit added to the olive oil, could contribute to the creation of an oleo-tourism experience.

## Figures and Tables

**Table 1:** Physicochemical characteristics of the olive oils.

Parameters		CO	C1	C2	AOMC1	AOMC2
Density		0.908 ± 0.005 ^a^	0.915 ± 0.002 ^aA^	0.917 ± 0.002 ^aA^	0.914 ± 0.005 ^aA^	0.916 ± 0.002 ^aA^
Colour	L	21.810 ± 0.043 ^a^	18.370 ± 0.036 ^bA^	20.223 ± 0.020 ^cC^	10.826 ± 0.005 ^dB^	10.245 ± 0.034 ^eD^
	a*	−0.843 ± 0.015 ^a^	−0.843 ± 0.661 ^aA^	−0.378 ± 0.021 ^bC^	0.226 ± 0.010 ^cB^	0.540 ± 0.122 ^dD^
	b*	13.503 ± 0.047 ^a^	11.393 ± 0.072 ^bA^	12.756 ± 0.083 ^cC^	2.483 ± 0.156 ^dB^	2.606 ± 0.176 ^dD^
FFA (%)		0.175 ± 0.007 ^a^	0.185 ± 0.005 ^aA^	0.225 ± 0.001 ^bC^	0.380 ± 0.012 ^cB^	0.410 ± 0.014 ^dD^
PV (meq/kg)		11.208 ± 0.325 ^a^	11.542 ± 0.477 ^aA^	12.458 ± 0.409 ^aC^	15.645 ± 0.161 ^bB^	17.865 ± 0.163 ^cD^
K_232_		1.651 ± 0.001 ^a^	1.928 ± 0.008 ^bA^	2.245 ± 0.001 ^cC^	2.135 ± 0.010 ^dB^	2.889 ± 0.014 ^eD^
K_270_		0.136 ± 0.006 ^a^	0.210 ± 0.003 ^bA^	0.249 ± 0.014 ^cC^	0.256 ± 0.002 ^cB^	0.262 ± 0.007 ^cC^
Chlorophylls (mg/kg)		6.056 ± 0.010 ^a^	4.124 ± 0.040 ^bA^	4.371 ± 0.013 ^cC^	2.712 ± 0.029 ^dB^	1.984 ± 0.019 ^eD^
Carotenoïds (mg/kg)		1.786 ± 0.028 ^a^	1.874 ± 0.010 ^aA^	1.960 ± 0.002 ^bC^	1.284 ± 0.040 ^cB^	1.048 ± 0.029 ^dD^
Total phenols (mg GAE/kg)		385.703 ± 0.020 ^a^	517.880 ± 0.030 ^bA^	668.720 ± 0.040 ^cC^	451.620 ± 0.010 ^dB^	587.640 ± 0.010 ^eD^

Different lowercase letters (a,b,…) in the same row compare the effect of concentration and indicate significant differences at (*p* < 0.05). Different uppercase letters (A,B,…) in the same row compare the process (co-crushing vs. maceration) and indicate significant differences at (*p* < 0.05).

**Table 2:** Fatty acid profile of control and flavoured olive oils.

Fatty Acids	C0	C1	C2
Palmitic acid (C16:0)	18.048 ± 0.314 ^a^	17.871 ± 0.055 ^a^	17.809 ± 0.037 ^a^
Palmitoleic acid (C16:1)	2.291 ± 0.040 ^a^	2.270 ± 0.064 ^a^	2.237 ± 0.019 ^a^
Heptadecanoic acid (C17:0)	0.0349 ± 0.001 ^a^	0.035 ± 0.001 ^a^	0.035 ± 0.007 ^a^
Heptadecenoic acid (C17:1)	0.274 ± 0.353 ^a^	0.066 ± 0.001 ^a^	0.066 ± 0.000 ^a^
Stearic acid (C18:0)	2.217 ± 0.028 ^a^	2.211 ± 0.045 ^a^	2.230 ± 0.005 ^a^
Oleic acid (C18:1)	60.434 ± 0.278 ^a^	60.512 ± 0.201 ^a^	60.576 ± 0.045 ^a^
Linoleic acid (C18:2)	15.821 ± 0.015 ^a^	15.939 ± 0.054 ^b^	15.946 ± 0.020 ^b^
Linolenic acid (C18:3)	0.564 ± 0.006 ^a^	0.553 ± 0.015 ^ac^	0.546 ± 0.002 ^bc^
Arachidic acid (C20:0)	0.360 ± 0.022 ^a^	0.377 ± 0.006 ^ac^	0.390 ± 0.006 ^bc^
Eicosenoic acid (C20:1)	0.147 ± 0.015 ^a^	0.151 ± 0.010 ^ab^	0.154 ± 0.010 ^ac^
Saturated fatty acid (SFA)	20.670 ± 0.266 ^a^	20.505 ± 0.082 ^a^	20.473 ± 0.046 ^a^
Monounsaturated fatty acid	63.148 ± 0.507 ^a^	63.002 ± 0.125 ^a^	63.034 ± 0.029 ^a^
Polyunsaturated fatty acid	16.386 ± 0.019 ^a^	16.493 ± 0.069 ^ab^	16.493 ± 0.022 ^b^

Different letters in the same row indicate significant differences at (*p* < 0.05).

**Table 3:** Colour parameters L, a* and b* of EVOOs during storage.

Storage (days)	Olive Oil Samples	L	a*	b*
0	C0	21.81 ± 0.043 ^cA^	−0.843 ± 0.015 ^bA^	13.503 ± 0.047 ^cA^
	C1	18.37 ± 0.036 ^aA^	−0.842 ± 0.018 ^bA^	11.393 ± 0.072 ^aA^
	C2	20.223 ± 0.02 ^bA^	−0.378 ± 0.021 ^aA^	12.756 ± 0.083 ^bA^
21	C0	20.247 ± 0.05 ^cB^	−0.833 ± 0.015 ^cA^	12.71 ± 0.026 ^bB^
	C1	18.310 ± 0.017 ^aA^	−0.743 ± 0.05 bB	10.176 ± 0.1 ^aB^
	C2	19.793 ± 0.032 ^bB^	−0.443 ± 0.02 ^aB^	12.703 ± 0.05 ^bA^
35	C0	18.417 ± 0.308 ^bC^	−0.89 ± 0.026 ^cA^	11.66 ± 0.049 ^bC^
	C1	18.330 ± 0.01 ^bA^	−0.740 ± 0.04 ^bB^	10.436 ± 0.04 ^aC^
	C2	17.146 ± 0.09 ^aC^	−0.483 ± 0.037 ^aB^	10.243 ± 0.096 ^aB^
56	C0	17.42 ± 0.073 ^aD^	−0.96 ± 0.01 ^aB^	10.81 ± 0.04 ^bD^
	C1	17.646 ± 0.065 ^aB^	−0.746 ± 0.030 ^bB^	9.726 ± 0.032 ^aD^
	C2	17.873 ± 0.047 ^aD^	−0.66 ± 0.036 ^cC^	10.256 ± 0.023 ^bB^
83	C0	16.556 ± 0.037 ^aE^	−0.996 ± 0.077 ^cB^	9.870 ± 0.098 ^aE^
	C1	16.113 ± 0.156 ^aC^	−0.849 ± 0.031 ^bA^	9.700 ± 0.01 ^aD^
	C2	17.723 ± 0.085 ^bE^	−0.76 ± 0.026 ^aD^	9.870 ± 0.05 ^aC^
98	C0	14.286 ± 0.031 ^aF^	−1.253 ± 0.049 ^aC^	8.543 ± 0.04 ^aF^
	C1	15.653 ± 0.23 ^bD^	−1.063 ± 0.015 ^bC^	9.2130.025 ^bE^
	C2	15.986 ± 0.058 ^bF^	−0.85 ± 0.045 ^cE^	9.193 ± 0.051 ^bC^
130	C0	12.903 ± 0.04 ^aG^	−1.153 ± 0.02 ^aD^	7.826 ± 0.056 ^aG^
	C1	12.276 ± 0.066 ^aE^	−1.09 ± 0.065 ^aC^	8.903 ± 0.055 ^bF^
	C2	12.166 ± 0.065 ^aG^	−0.93 ± 0.016 ^bF^	8.866 ± 0.071 ^bD^
165	C0	11.800 ± 0.05 ^aH^	−1.568 ± 0.015 ^cE^	7.238 ± 0.034 ^aH^
	C1	11.230 ± 0.012 ^aF^	−1.219 ± 0.011 ^bD^	8.842 ± 0.021 ^bF^
	C2	11.010 ± 0.013 ^aH^	−1.02 ± 0.026 ^aF^	8.012 ± 0.011 ^aE^

Different lowercase letters (a,b,…) in the same column for the same storage duration indicate significant differences, at (*p* < 0.05). Different uppercase letters (A,B,…) in the same column for the same oil at different storage durations indicate significant differences, at (*p* < 0.05).

**Table 4:** Main characteristics of study participants (224 people).

Variables	Levels	N	%
Age	<25	102	45.5
	25–40	76	33.9
	40–60	44	19.6
	>60	2	1
Gender	F	122	54.5
	M	102	45.5
	Other	0	0
Educational level	Bachelor’s degree	38	17
	High school diploma	116	51.8
	Lower secondary school certificate	18	8
	Master’s degree	43	19.2
	PhD or other	9	4

**Table 5:** Habit of olive oil consumption, knowledge of aromatised EVOOs and cloves and purchase intention.

Question	Levels	%
How do you buy your olive oil?	Directly from the producer	86.5
	From the supermarket	4
	From the grocer’s	3.6
	From the market	5.9
On average, how often do you use olive oil in your personal diet?	3 times/week	70.2
	1 to 2 times/week	15.4
	1 time/15 days	8.7
	Less	5.7
On average, what is your household’s monthly consumption of olive oil?	<½ L/month	7.8
	1 L/month	27.2
	2 L/month	23.3
	>2 L/month	41.7
Use of olive oil	Salad dressing	51.5
	Cooking	45.6
	Frying	2.9
Do you know about flavoured oils for food uses?	Yes	30
	No	70
What would be your reservations about buying ready-to-use flavoured oils in supermarkets?	Lack of advice	22.3
	No specialised point of sale	15.5
	Doubts about the quality of the product	46.6
	The price is higher than that of an unflavoured oil	15.5
Do you know the health benefits of cloves?	Yes	52.7
	No	47.3
If you find a clove-flavoured olive oil, would you be willing to buy it?	Yes	83.9
	No	14.3
	Probably	1.8

## Data Availability

The original contributions presented in this study are included in the article/Supplementary Materials. Further inquiries can be directed to the corresponding author.

## Supplementary Materials

The following supporting information can be downloaded at: https://www.mdpi.com/article/10.3390/foods14122114/s1, Table S1: Changes in free fatty acidity (FFA), K232, K270, chlorophylls, carotenoids and total phenols during storage at 60 °C.
