# Supplementary material for: Flavouring Tunisian Extra Virgin Olive Oil (EVOO) with Cloves: Quality Indices, Stability, and Consumers’ Purchase Survey
Source: Foods. 2025 Jun 16;14(12):2114. doi: 10.3390/foods14122114 (PMC12192215; doi:10.3390/foods14122114)
Supplement: Supplementary file 1 [file foods-14-02114-s001.zip › foods-3629017-supplementary.pdf]

foods

MDPI

Supplementary material:

Table S1: Changes in free fatty acidity (FFA), $K_{232}$, $K_{270}$, chlorophylls, carotenoids and total phenols during storage at 60°C

| Storage (days) | Olive oil samples | FFA (%) | $K_{232}$ | $K_{270}$ | Chorophylls (mg/kg) | Carotenoids (mg/kg) | Total phenolics (mg GAE/kg) |
|---|---|---|---|---|---|---|---|
| 0 | C0 | 0.175±0.01a | 1.652±0.10a | 0.136±0.02a | 6.056±0.07a | 1.786±0.03a | 385.703±5.601a |
| | C1 | 0.185±0.01b | 1.928±0.05b | 0.211±0.03b | 4.123±0.06b | 1.874±0.01a | 517.88±13b |
| | C2 | 0.225±0.01c | 2.245±0.06c | 0.281±0.03c | 4.371±0.06b | 1.960±0.02a | 668.72±14c |
| 21 | C0 | 0.44±0.01a | 1.789±0.09a | 0.175±0.02a | 4.193±0.07a | 1.435±0.09a | 322.349±13.383a |
| | C1 | 0.485±0.01b | 1.909±0.07a | 0.243±0.01b | 3.707±0.04a | 1.316±0.02a | 511.995±16.619b |
| | C2 | 0.57±0.03c | 2.127±0.04b | 0.330±0.03c | 3.970±0.09a | 1.102±0.03b | 650.317±15.155c |
| 35 | C0 | 0.61±0.02a | 1.719±0.03a | 0.207±0.01a | 3.646±0.16a | 1.198±0.02a | 283.270±13.083a |
| | C1 | 0.60±0.02a | 1.862±0.06b | 0.282±0.02b | 3.490±0.01a | 1.216±0.01a | 498.948±17.380b |
| | C2 | 0.66±0.01b | 2.088±0.07c | 0.363±0.01c | 3.456±0.07a | 1.021±0.02b | 605.933±19.244c |
| 56 | C0 | 0.92±0.01a | 1.728±0.05a | 0.296±0.01a | 3.040±0.17a | 0.970±0.02a | 265.425±12.99a |
| | C1 | 0.935±0.02a | 1.962±0.08a | 0.341±0.05a | 3.260±0.05a | 1.010±0.08a | 470.295±17.44b |
| | C2 | 0.89±0.02a | 2.243±0.10b | 0.381±0.02b | 3.283±0.07a | 0.891±0.03a | 593.146±25.92c |
| 83 | C0 | 1.31±0.02a | 1.770±0.02a | 0.360±0.01a | 2.714±0.18a | 0.944±0.03a | 234.006±14.42a |
| | C1 | 1.29±0.02a | 1.916±0.02a | 0.436±0.03b | 3.124±0.21a | 0.798±0.02a | 352.406±15.41b |
| | C2 | 1.185±0.03b | 2.219±0.05b | 0.489±0.04b | 3.252±0.06a | 0.889±0.01a | 573.513±24.69c |
| 98 | C0 | 1.63±0.01a | 1.775±0.1a | 0.442±0.01a | 2.239±0.06a | 0.782±0.02a | 223.420±12.003a |
| | C1 | 1.615±0.01a | 1.982±0.06a | 0.501±0.03b | 2.479±0.1a | 0.798±0.07a | 321.057±15.506b |
| | C2 | 1.67±0.02b | 2.150±0.01a | 0.504±0.03b | 3.155±0.04b | 0.873±0.03a | 552.183±17.806c |
| 130 | C0 | 2.215±0.06a | 2.025±0.03a | 0.605±0.01a | 1.413±0.16a | 0.470±0.05a | 213.833±13.875a |
| | C1 | 2.425±0.02b | 2.154±0.04a | 0.545±0.03b | 2.114±0.14b | 0.669±0.06b | 258.995±15.194b |
| | C2 | 2.210±0.03a | 2.246±0.05a | 0.532±0.01b | 2.481±0.07b | 0.687±0.01b | 401.93±25.010c |
| 165 | C0 | 3.110±0.01a | 2.483±0.04a | 0.702±0.01a | 0.923±0.06a | 0.354±0.04a | 207.446±10.054a |
| | C1 | 3.290±0.01b | 2.221±0.03b | 0.557±0.01b | 1.542±0.04b | 0.500±0.01a | 215.203±9.338a |
| | C2 | 3.235±0.03b | 2.963±0.09c | 0.542±0.01b | 1.861±0.06b | 0.481±0.03a | 382.753±24.002b |

Different letters in lowercase form (a,b,..) at the same column for the same storage duration indicate significantly differences at ($p<0.05$)

Different letters in uppercase form (A, B,..) at the same column for the same oil at different storage duration indicate significantly differences at ($p<0.05$)
